# Long-term results of middle fossa plugging of superior semicircular canal dehiscences: clinically and instrumentally demonstrated efficiency in a retrospective series of 16 ears

**DOI:** 10.1007/s00405-015-3715-5

**Published:** 2015-07-24

**Authors:** Hans Thomeer, Damien Bonnard, Vincent Castetbon, Valérie Franco-Vidal, Patricia Darrouzet, Vincent Darrouzet

**Affiliations:** Department of Otolaryngology and Skull Base Surgery, Pellegrin University Hospital, Bordeaux Segalen University, 33000 Bordeaux, France; Department of Otorhinolaryngology, University Medical Center Utrecht, 85500, Heidelberglaan 100, 3508 GA Utrecht, The Netherlands

**Keywords:** Hearing impairment, SCDS, Semicircular canal, Dehiscence, Middle fossa, Surgery, Vertigo, Tinnitus

## Abstract

The objective of this study is to report the surgical outcome after middle fossa approach (MFA) plugging in patients suffering from a superior semi-circular canal dehiscence (SCD) syndrome. This is a retrospective case review. Tertiary referral center. Sixteen ears in 13 patients with a SCD syndrome suffering from severe and disabling vestibular symptoms with a bony dehiscence on CT scan >3 mm and decreased threshold of cervical vestibular evoked potentials (cVEMPs). We assessed preoperatively: clinical symptoms, hearing, cVEMPs threshold, size of dehiscence and videonystagmography (VNG) with caloric and 100 Hz vibratory tests. Postoperatively, we noted occurrences of neurosurgical complication, evolution of audiological and vestibular symptoms, and evaluation of cVEMP data. Tullio’s phenomenon was observed in 13 cases (81.3 %) and subjectively reported hearing loss in seven (43.7 %). All patients were so disabled that they had to stop working. No neurosurgical complications were observed in the postoperative course. In three cases (16.6 %), an ipsilateral and transitory immediate postoperative vestibular deficit associated with a sensorineural hearing loss (SNHL) was noted, which totally resolved with steroids and bed rest. All patients were relieved of audiological and vestibular symptoms and could return to normal activity with a mean follow-up of 31.1 months (range 3–95). No patient had residual SNHL. cVEMPs were performed in 14 ears postoperatively and were normalized in 12 (85.7 %). Two of the three patients operated on both sides kept some degree of unsteadiness and oscillopsia. MFA plugging of the superior semi-circular canal is an efficient and non-hearing deteriorating procedure.

## Introduction

The superior semicircular canal dehiscence (SCD) syndrome is evoked when hyperacusis, Tullio’s phenomenon, autophony, oscillopsia, pressure-induced vertigo, otosclerosis-like mixed hearing loss and pulsatile tinnitus are encountered, isolated or associated in very different clinical patterns [1]. In the event of mixed hearing loss, preservation of acoustic reflexes and decreased bone conduction (BC) threshold on low frequencies (<1 kHz) help to differentiate SCD syndrome from otosclerosis [2–4]. A positive diagnosis depends on high-resolution computed tomography (HRCT) scan, but the rate of false-positive image outcome should be considered [5] and further evidence may be necessary in some settings. Cervical and air-conducted ocular vestibular evoked myogenic potentials (c- and oVEMPs) have shown a high sensitivity in surgically demonstrated SCD and help to validate as true positive some bony gaps observed in imaging studies [5–11]. While a consensus has emerged on how to explore SCD syndrome patients, the right way to manage them is still debatable. Which patients are to be operated upon? Should the canal be resurfaced, capped or plugged? Which material is to be used for plugging? Should plugging or resurfacing be conducted by the transmastoid or middle fossa approach (MFA)? The former seems more familiar and secure for otolaryngologists [12–16], while the latter is more logical and efficient from the pathophysiological point of view for neurotologists [1, 9, 17, 18]. The respective defenders of the two approaches have published an increasing number of papers in recent years. Like us, most authors perform surgery only in patients highly disabled by vestibular symptoms [1, 9, 12, 19, 20], though others believe it might be useful even in patients only affected by audiological symptoms (isolated pulsatile tinnitus, hearing loss, autophony, hyperacusis) [13, 14, 19].

The objective of this retrospective report is to report on the results in 16 new ears treated by MFA canal plugging thereby providing insight into the risks and efficiency of this technique. An algorithm for decision-making is also provided.

## Materials and methods

### Population and data analysis

The charts of patients referred to our tertiary Otolaryngology and Skull Base Department and operated on by MFA of SCD by the senior author (VD) between 2006 and 2013 were retrospectively assessed and listed in a database (Excel, Microsoft, USA). Inclusion criteria were as follows: a/patients suffering from severe incapacitating balance problems; b/a positive HRCT with an SCD >3 mm in the Pöschl plane; c/a decreased threshold (<90 dB) of cVEMPs. Diagnosis of SCD syndrome was made after a thorough search for a history of head trauma, an otoscopic and physical examination, and after obtaining clinical data concerning audiological and vestibular symptoms. We looked for Hennebert’s and Halmagyi’s signs. Preoperatively, a complete otoneurological work-up was performed, including:

- a tone and speech audiogram with search for supranormal BC on low frequencies and calculation of: a/pure tone average (PTA) with air conduction (AC) thresholds according to the American Academy of Otolaryngology—Head and Neck Surgery hearing classification system to describe functional outcome (0.5, 1, 2 and 4 kHz) [40]; b/PTA at low frequencies (0.25, 0.5 and 1 kHz); c/air bone gap (ABG, mean 0.5, 1, 2 and 4 kHz). The speech reception threshold (SRT) was calculated with dissyllabic words.
- a tympanometry with search for acoustic reflexes and pressure-induced vertigo.
- a computerized videonystagmography (VNG) with caloric testing and vibratory test at 100 Hz (Ulmer’s System, Synapsis, Marseilles, France).
- click-evoked cVEMPs, with calculation of thresholds and amplitudes on both sides. cVEMPs were obtained with clicks of 500 Hz in descending thresholds from 110 dB SPL to 60 dB on both ears (Synapsis, Marseilles, France). cVEMPs were analyzed as abnormal when the threshold was <90 dB. In all subjects, cVEMPs were compared to the contralateral side, which could also appear dehiscent. In cases of bilateral SCD, the most pathological side was operated first.
- On HRCT, the position and size of the dehiscence was calculated on the reformatted slices in Pöschl’s plane.

The postoperative course was analyzed by recording the following:

- any immediate complications due to the neurosurgical approach (cerebrospinal fluid leak, meningitis, facial paralysis, intracranial hematoma, seizure, wound superinfection) or to acute or delayed labyrinthine suffering, i.e., sensorineural hearing loss (SNHL), tinnitus, vertigo, nystagmus contralateral to operated side
- hospital and intensive care unit (ICU) stay duration
- audiometric data obtained at D7

When necessary and possible, patients were managed postoperatively by physiotherapists to accelerate their vestibular compensation.

At 1 month postoperative, the following were performed:

- a clinical evaluation of audio-vestibular symptoms
- a complete audiogram
- a cVEMP evaluation

If the follow-up was >6 months, we also retained the last recorded audiogram or cVEMPs.

Data analysis was performed with statistical software (SPSS Version 19.0). Pre- and postoperative hearing thresholds were compared as well as ABG. Quantitative variables were compared with an unpaired Student’s *t* test. Qualitative variants were compared with the *χ*^2^ test or the Fisher test for small numbers. The level of statistical significance was reached when *p* < 0.05.

### Surgical technique

A modified MFA was used in all ears. After an 8 cm skin incision running vertically from the tragus, a 4 by 4 cm bone flap was cut vertical to the external auditory canal. The dura mater was gently elevated from the middle fossa plate using blunt instruments pushing cottonoids forward and laterally. No retractor was used in order to minimize the extradural retraction of the temporal lobe. Bipolar coagulation induced retraction of the dura mater and improved exposure of the bony surface. CSF leak and bleeding were cautiously avoided thanks to the use of cottonoids and Surgicel^®^. As much as possible, the SCD was sought only when a dry operative field free of blood was obtained in order to avoid suctioning in its vicinity. When identified, the SCD was immediately plugged with bone wax and then covered with bone paté. Finally, a fascia temporalis patch was draped on the petrous bone and secured with 2 ml of fibrin glue (Tisseel, Baxter, USA). The numerous tegmental dehiscences often observed in the roof of the petrous bone were addressed and closed during the same surgical procedure with bone paté and fascia. The bone flap was put back after the dura mater had been attached by two silk sutures. The patient stayed for at least 24 h in an ICU for neurological monitoring.

## Results

### Patient description

During this period, 58 patients with a SCD syndrome meeting our three-criteria definition were explored. Of these, 15 (18 ears, 31 %) were surgically managed. Two patients were not included in this series: one had been previously operated in another center by a transmastoid approach and the second had a history of an ipsilateral sphenoid wing meningioma operated via MFA and was therefore also managed by a transmastoid approach. Finally, a total of 13 patients (16 ears) were included in the study. All patient characteristics are listed in Table 1. None had undergone any previous ear surgery. Their mean age was 47.3 years [standard deviation (SD) 7.5; range 28–61]. Five were male. There were six left and ten right ears. Mean delay between referral and surgery was 5.2 months (range 1–24). Mean size of dehiscence was 4.4 mm (range 3.0–5.5 mm; SD 1.15). Six patients had unilateral SCD and six had bilateral SCDs on HRCT, but only three had bilateral SCD syndrome that met our criteria (abnormal CT, symptoms and abnormal cVEMPs). These were operated on both sides: in two cases, the SCD syndrome was initially bilateral and they were operated on the opposite side after a short delay: 8 and 28 months for the first (ears 2–3) and the second one (ears 8–9). In the latter, bilateralization of disease took almost 5 years and the delay between interventions was 61 months. In seven ears (43.7 %), we noted a previous history of head trauma: mean delay between trauma and first visit was 137 months (SD 173.3; range 12–360 month). A subjectively reported hearing loss was noted in seven ears (43.7 %). Otoscopy was normal in all of these patients.

**Table 1:** Patient Characteristics with SCD

Case	1	2^a^	3^a^	4	5	6	7	8^b^	9^b^	10	11	12^c^	13^c^	14	15	16
Age/sex	39/M	50/M	55/M	47/F	22/M	53/F	46/F	58/F	61/F	45/F	51/F	44/F	44/F	57/F	28/M	44/M
Laterality	B	B	B	B	U	B	B	B	B	B	U	B	B	U	U	B
Side	R	R	L	L	L	L	L	R	L	L	R	R	L	L	R	L
HL	−	−	−	+	−	+	+	−	−	+	+	−	−	+	+	−
Tinnitus	+	+	+	+	+	+	+	−	−	+	+	+	+	+	+	−
Aural Fullness	+	−	−	−	−	−	+	−	−	−	+	−	−	+	+	+
Autophony	−	+	+	+	−	−	−	−	−	+	−	+	+	+	+	+
Vertigo	+	+	+	+	+	+	+	+	+	+	+	−	−	−	−	+
Instability	+	+	+	+	+	+	+	+	+	+	+	+	+	−	+	+
Hennebert S	+	+	+	−	+	−	−	+	+	−	NA	+	+	−	+	+
Tullio Ph	−	+	+	+	+	+	+	+	+	+	NA	+	+	−	+	+
cVEMP_pre (dB)	70	90	70	70	80	<90	80	NA	80	70	NA	90	70	80	70	70
cVEMP_post (dB)	80	100	100	100	90	100	90	NA	NA	100	NA	90	90	90	100	90
VNG	NA	NA	NA	vertNyst	NA	NA	hypoR^d^	Normal	Normal	vertNyst	NA	vertNyst	vertNyst	Normal	vertNyst	NA
SRT preop	27	22	35	25	10	25	15	25	25	25	42	20	20	15	15	10
SRT postop	7	27	40	50	30	45	30	40	55	75	35	15	20	20	NA	20
SRT final	7	27	25	25	15	17	10	25	35	30	35	20	15	15	10	10
Delay (Mth)	3	2	9	5	3	24	9	4	4	2	1	4	6	1	3	4
DOH (ICU)	7 (2)	11 (8)	8 (4)	8 (2)	8 (3)	13 (2)	8 (2)	4 (2)	4 (3)	8 (2)	10 (2)	7 (3)	7 (2)	7 (2)	7 (2)	7 (2)

*NA* not applicable, *B/U* bi-or unilateral affected patient, *R/L* right or left ear, *hypoR* hyporeflexia, *aR* areflexia, *Mth* month, *vertNyst* vertical nystagmus during vibratory test, *DOH* days of hospitalization, *ICU* intensive Care Unit

Three patients were operated on both sides (indicated with ^a, b, c^ respectively)

^d^contralateral hyporeflexia

A total of 81.3 % (13 out of 16 ears) reported tinnitus of which most (12 of 13) were pulsatile.

The first patient operated did not undergo cVEMPs threshold measurement (case 11) since it was not routinely performed at that time. However, the cVEMPs amplitude at 100 dB was abnormally wide on the operated side compared to contralateral one. In the 14 ears, cVEMPs thresholds <90 dB were obtained preoperatively. In one case, they were not available owing to technical problems (case 9).

A VNG was performed in 9 ears (56.2 %). It showed a caloric deficit >20 % in three ears (33.3 %) and a vibration-induced vertical nystagmus in five (66.7 %; ears 4, 10, 12, 13 and 15).

### Postoperative evolution

**Fig. 1:**
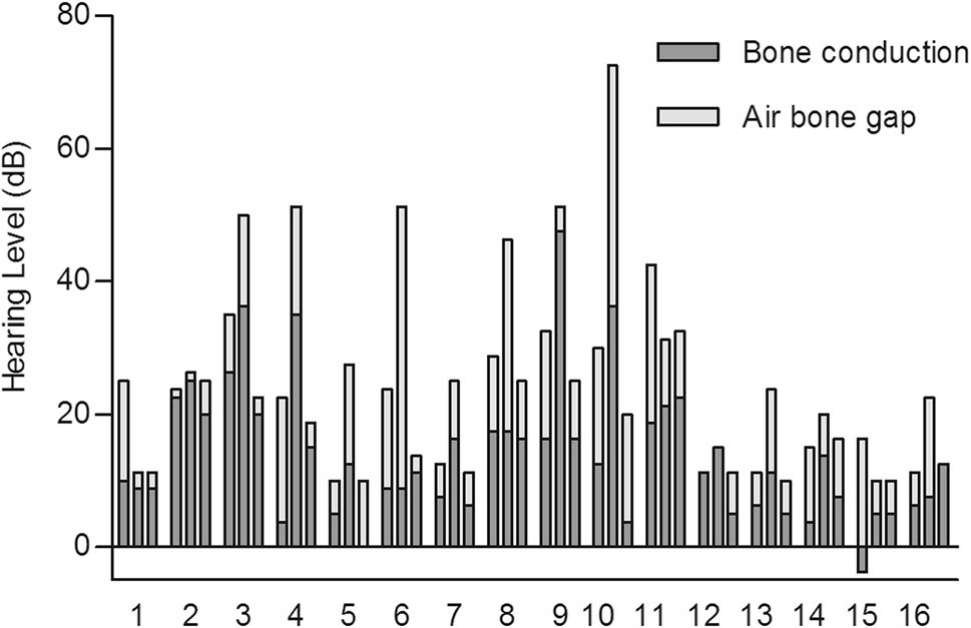
Individual preoperative, postoperative and most recent hearing levels for bone conduction and air conduction in 16 operated ears. First column of each ear indicates preoperative hearing threshold (according to the AAO-HNS criteria), second column indicates direct postoperative results (mostly day 6–7 postoperatively) and third column indicates threshold obtained at last audiogram performed

**Fig. 2:**
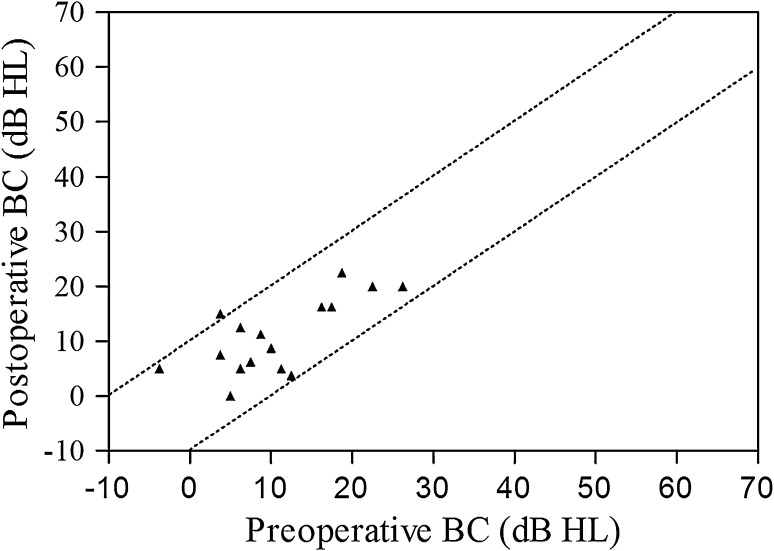
Individual postoperative audiometric outcome according to Amsterdam Hearing Evaluation Plots (AHEPs). The *two dotted diagonal lines* enclose the area within bone conduction that changed by less than 10 dB. Only one case of mild postoperative deterioration in bone conduction of more than 10 dB was found (case 4, pre- and postoperative BC, respectively, 3.8 and 15 dB); *BC* bone conduction, *dB HL* decibel hearing level

No postoperative complication was observed. Mean global hospital stay was 7.8 days (SD 2.2, range 4–13) and mean ICU stay was 2.7 days (SD 1.5, range 2–8). The mean follow-up was 31.1 months (Median 23.0 and SD 26.8; range 3–95). Preoperative, immediate postoperative and most recent AC, BC, PTA and ABG levels are reported in Table 1. No patient had any residual SNHL. Three (ears 4, 9 and 10) had a mild postoperative SNHL (mean BC were 35, 47.5 and 36.3 dB, respectively), associated with an ipsilateral and transitory immediate vestibular deficit, which totally resolved with steroid taper and vasodilators (mean BC 15, 16.3 and 3.8 dB at last evaluation, respectively). The postoperative CT scan was normal in these patients apart from a pneumolabyrinth. An overview of pre- and postoperative BC according to the Amsterdam Hearing Evaluation Plot [15, 16] is shown in Figs. 1 and 2. Considering speech audiometry results, mean preoperative SRT was 22.3 dB (SD 8.5) while the immediate postoperative value was 33.9 dB (SD 17.6) and the final level 20.1 dB (SD 9.0) (Table 1). The difference between pre- and postoperative SRT was not significant (*p* > 0.05). All patients were relieved of their pulsatile tinnitus. In Table 2, the distribution of hearing outcome is shown, according to respective moment of measure (no significant outcome was encountered).

**Table 2:** Audiometric Results (dB)

Type	Low Frequencies PTA (0.25–0.5–1.0)	PTA (0.5–1.0–2.0–4.0)
AC gain	7.4 (SD 7.7)	4.5 (SD 5.3)
AC preop	23.6 (SD 12.1)	21.7 (SD 10.1)
AC postop	17.1 (SD 7.1)	17.2 (SD 7.1)
BC loss	2.5 (SD 7.2)	0.2 (SD 5.6)
BC preop	6.1 (SD 7.9)	10.8 (SD 7.8)
BC postop	8.5 (SD 6.4)	10.9 (SD 6.8)
ABG reduction	9.0 (SD 10.9)	4.7 (SD 6.9)
ABG preop	17.5 (SD 12.1)	10.9 (SD 6.9)
ABG postop	8.5 (SD 6.1)	6.3 (SD 4.0)

Results are mean values of all cases (*N* = 16)

*SD* standard deviation, *AAO-HNS* American Academy of Otolaryngology—Head and Neck Surgery, *dB* decibel, *AC* air conduction, *BC* bone conduction, *ABG* air-bone gap, *preop* preoperative, *postop* postoperative

**Fig. 3:**
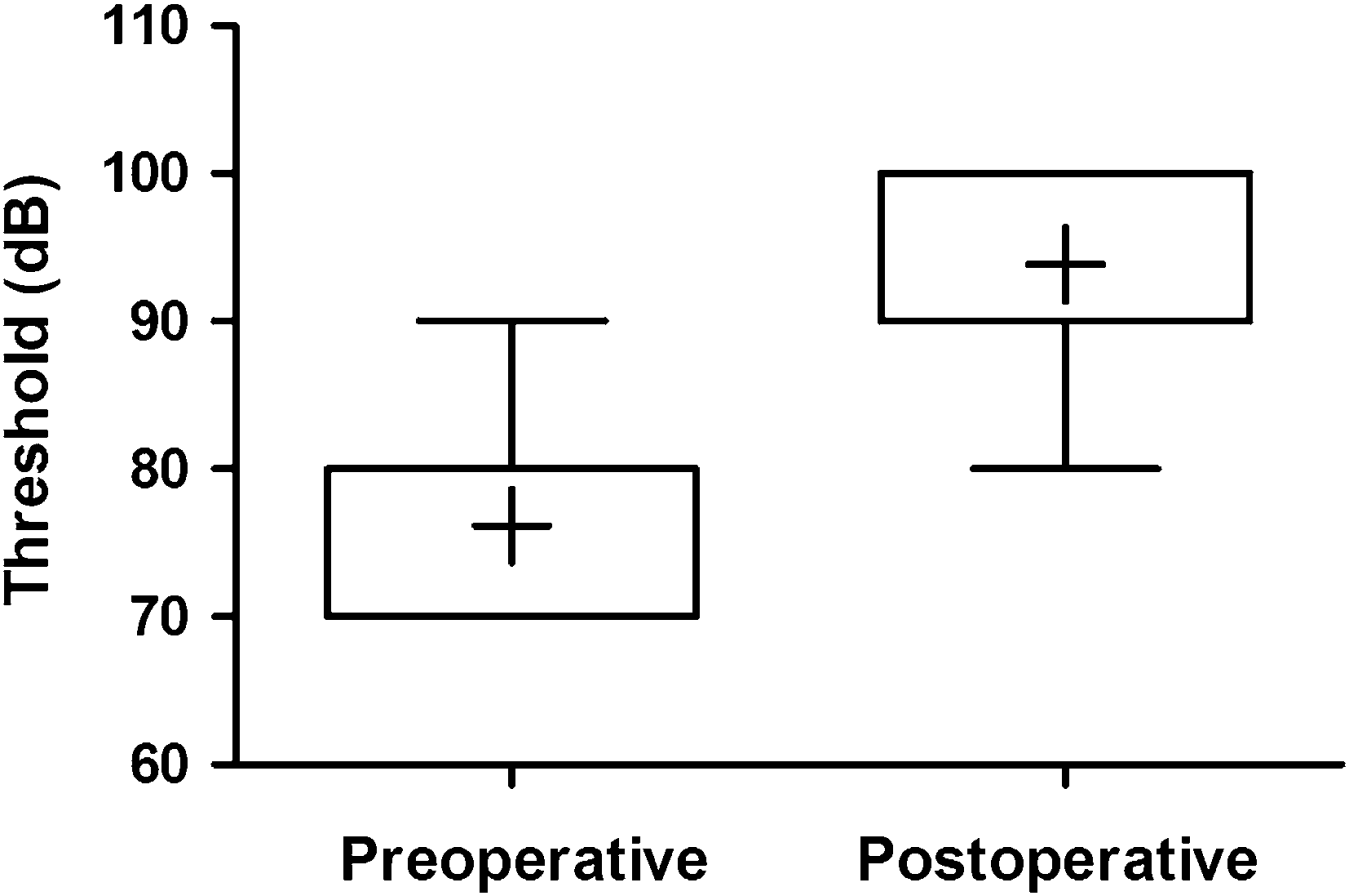
cVEMP Box Plot pre- and postoperatively. *Box* and *Whisker Plots* showing the difference in threshold between pre- and postoperative cVEMP testing (*p* value 0.0021). *Box* median and IQR (interquartile range), *Plus* mean value, *Whiskers* range

Postoperative evolution of cVEMPs was the following: mean threshold increased from 76.1 to 94.4 dB (*p* = 0.0021). In all ears except two, thresholds were normalized and amplitudes returned to normal. In ears 9 and 11, cVEMPs were not performed after surgery. Figure 3 demonstrates a Box Whisker Plot showing a large increase in cVEMPs after surgery, although the small sample size did not allow this difference to be significant (*p* = 0.34).

Overall, 12 of the 13 patients (92.3 %) returned to their normal daily activity and were relieved of their symptoms (14 of 16 ears). Two patients who were operated bilaterally still experienced some oscillopsia when walking (ears 2–3 and 8–9). During follow-up, 7 of the 13 patients received postoperative vestibular rehabilitative treatment, including those operated bilaterally.

## Discussion

Our diagnostic criteria for SCD syndrome were and still are very restrictive, since we only considered cases with abnormal HRCT scans and decreased cVEMPs thresholds. This philosophy might be criticized but has the advantage of excluding “nearly dehiscent” patients in whom the surgical outcome is difficult to analyze, even if surgery may be efficient in this pathologic entity [17, 18]. Moreover, this homogeneous series only included patients with incapacitating vestibular symptoms, even if a considerable amount of them complained of associated hearing disorders. In our center, we do not consider surgery in the event of isolated non-vestibular symptoms such as pulsating tinnitus or mixed hearing loss. All the operated ears suffered from a dura mater-covered SCD. No case of direct venous sinus-SC contact was included, which is in contrast with other series [21–23]. Our series includes only 27.6 % of the 58 cases diagnosed in our center since 2006, the great majority only being monitored. The large series in the literature are less homogeneous and include patients in whom surgery was indicated for less incapacitating symptoms like mixed hearing loss or pulsatile tinnitus [13, 14, 19]. In these, the disease might be less advanced and more focal, thereby reducing the risk of residual disability. Interestingly, Niesten et al. [24] observed in a cohort of 104 patients that auditory symptoms were merely associated with larger dehiscences that were closer to the ampulla than those with only vestibular symptoms.

Our series covers a fairly long experience of this surgery, our first patient having been operated on 8 years ago. Consequently, our follow-up is much longer than in other reports where the ranges were from 3 to 15 months [13, 18, 25]. The length of follow-up is of paramount importance when evaluating the surgical treatment of a disease that may become bilateral and above all relapse. The clinical symptoms observed in our patients are similar to those reported in the literature [9, 13, 18, 19]. One fifth of the patients were operated bilaterally. This is in agreement with the assumption that the most probable etiology of this pathology is congenital or due to a developmental disorder of the tegmental bone in early life, with a resulting thin bilateral layer of the bony middle fossa [19, 26]. The role of a subsequent traumatic event is apparent in the pathogenesis of this syndrome since we observed this in one third of our patients. As demonstrated by ears 8 and 9 (same patient), some patients may develop symptoms on the contralateral side after a delay of several years, even if the SCD was initially detected at HRCT without symptoms or abnormal cVEMPs. Initially, this patient had a bilateral SCD but an unilateral disease.

The results of the preoperative instrumental work-up deserve discussion. The 100 Hz vibration-induced vertical eye movement observed in five ears is one of the diagnostic arguments for the disease. One might wonder why this was not observed in all cases, as in the study by Aw et al. [27] where SCD patients were tested with precalibrated dual-search coils. In our opinion, the stimulation they used was more powerful. The caloric deficit observed in three ears might suggest that the disease is not limited to the canal and might involve other labyrinthine structures, especially in the most long-standing disabled patients. This vestibular deficit may explain why canal plugging is not immediately efficient on balance disorders and why postoperative balance rehabilitation is often necessary to obtain total vestibular symptom relief in these cases. We think that such instrumental vestibular evaluation is useful, its results being part of the prognostic factors for postoperative balance outcome.

Postoperative hearing outcome was merely satisfactory. We demonstrated that the transitory SNHL observed in three ears was not attributable to air bubbles. Other authors have observed this not uncommon phenomenon and attribute it to an inflammatory reaction to the plugging material [18, 20, 28]. Since it was observed at the beginning of our experience, it might also be a consequence of an inner ear trauma due to suctioning near the SCD during surgery. Therefore, we recommend a dry operative field before SCD exposure and plugging. The absence of residual SNHL in our series underlines the relatively atraumatic nature of MFA plugging. This could be due to the fact that plugging is performed far from the vestibule compared to transmastoid approaches in which the canal is opened close to the ampulla and the vestibule. With the latter, total SNHL has been reported [12, 29].

The ABG commonly observed in the low frequencies is known to be due to additional effects of supranormal BC and AC decrease [30]. We observed a noticeable improvement in this ABG with the dual effect of an AC decrease and a BC increase, particularly at the low frequencies. Seven of sixteen ears (43.7 %) reported hearing impairment preoperatively. This might be due to the fact that mostly low frequencies are affected, which functionally has a lesser impact on daily life. We recommend that low frequencies be used in order to obtain a more genuine surgical outcome.

cVEMP and more recently oVEMP testing have proven to be highly sensitive tests to objectify the third window phenomenon [9–11]. The postoperative normalization of cVEMP threshold we observed in our patients confirms the closure of the third windows and the normalization of inner ear hydraulics [9, 31, 32]. Regarding postoperative improvement of chronic imbalance and disequilibrium, various reports in the literature using the disability handicap inventory questionnaire (DHI) have shown a favorable outcome [33–35], whatever the technique used [34, 35]. The presented series did not utilize DHI questionnaires although self-perceived imbalance outcome in our cohort showed relief from symptoms (sound- and noise-induced vertigo, chronic imbalance) in most cases (12 of 13 patients, 92.3 %). Nonetheless, Janky et al. [33] using Head Impulse Tests (HIT) showed that surgical treatment induces global vestibular dysfunction that generally only impairs the superior semicircular canal on the operated side in the long term (>6 weeks postoperatively). Therefore, they recommend that all patients undergo a postoperative assessment of the risk of falling in order to avoid accidents immediately after surgery.

In the long term, SCD plugging has led to better results than canal resurfacing [19, 20, 25]. In contrast to fascia or bone powder, bone wax has been suspected experimentally and clinically to induce some degree of serous labyrinthitis and SNHL [25, 36]. In contrast to these reports, no patient in our cohort had any hearing sequelae. We doubt that bone wax induces long-term inner ear lesions and feel that semicircular canal plugging at the level of the dehiscence may induce minimal inner ear trauma by itself, as testified by SNHL cases reported by authors using other plugging materials such as bone paté with fibrin glue [12, 14] or fascia with bone paté [13, 18, 20]. Originally, MFA was advocated by Minor et al. as the default approach either to plug or resurface the SCD. Currently, however, this approach is criticized by otologists for its potentially lethal neurosurgical complications [13, 14, 17]. Experience in otoneurosurgical procedures is essential to practice it safely as we have done for decades in the management of posttraumatic facial paralysis and spontaneous tegmental CSF fistulas [37]. It has the great advantage of leading the surgeon to the exact location of the dehiscence without any risk of jeopardizing other parts of the labyrinth with a drill. We have modified the MFA to treat SCD by minimizing the retraction and dural elevation and focusing onto the arcuate eminence. The potential risks of the MFA led some otologists like Brantberg et al. [29] and Agrawal and Parnes [12] to use a transmastoid approach, which is more otological and more familiar to us. While there are potential neurosurgical complications of the MFA, we have never observed any in our long-term experience of it.

**Fig. 4:**
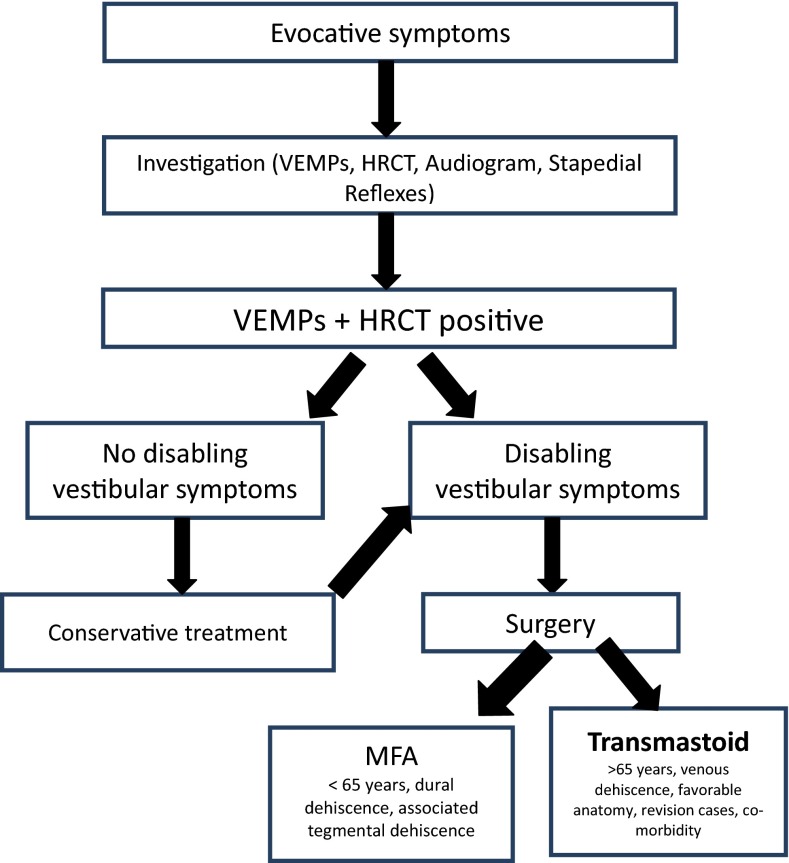
SCDS Algorithm

Since then, several small series of transmastoid plugging or resurfacing associated with short-term follow-up have been reported [12, 14–16, 38, 39]. These techniques lead to good results on audiological and vestibular symptoms but do not expose the SCD, unlike the MFA. Difficult access to the superior canal loop due to an overlying tegmen often necessitates dura mater coagulation/retraction. Moreover, the degree of temporal bone pneumatization may have a great impact on the success of the surgery as observed by Zhao et al. [13]. In addition, it does not allow resurfacing of the tegmental dehiscence, which is often associated with SCD, during the same surgical intervention. A higher likelihood of recurrence after plugging and a higher risk of total hearing loss due to double drilling of the canal near the vestibule exposes the patient to the potential risk of SNHL, as reported in the literature [12]. In our opinion, the transmastoid approach is indicated in MFA revision cases [19] and when the SCD syndrome is caused by direct contact with the superior petrosal sinus. It can also be considered when an associated tegmental dehiscence is associated or in poor general condition, in elderly patients (>65 year) and when an intracranial procedure is more at risk (anti-aggregant or anti-coagulant treatment). Finally, we have an algorithm to help clinicians in decision-making regarding treatment after the diagnosis of SCD syndrome (Fig. 4).

## Conclusion

This new series of surgically treated SCD patients demonstrates the good long-term efficiency and non-invasiveness of direct plugging by MFA. Patients were relieved of their disabling symptoms and the ABG resolved with no residual SNHL. These results should be kept in mind and compared in the future with those obtained with a purely transmastoid approach.

## References

[1] Minor LB, Solomon D, Zinreich JS, Zee DS (1998). Sound- and/or pressure-induced vertigo due to bone dehiscence of the superior semicircular canal. Arch Otolaryngol Head Neck Surg.

[2] Minor LB, Carey JP, Cremer PD, Lustig LR, Streubel SO, Ruckenstein MJ (2003). Dehiscence of bone overlying the superior canal as a cause of apparent conductive hearing loss. Otol Neurotol.

[3] Lehmann M, Ebmeyer J, Upile T, Sudhoff HH (2011). Superior canal dehiscence in a patient with three failed stapedectomy operations for otosclerosis: a case report. J Med Case Reports.

[4] Pritchett CV, Spector ME, Kileny PR, Heidenreich KD, El-Kashlan HK (2014). Surgical treatment of hearing loss when otosclerosis coexists with superior semicircular canal dehiscence syndrome. Otol Neurotol.

[5] Mondina M, Bonnard D, Barreau X, Darrouzet V, Franco-Vidal V (2013). Anatomo-radiological study of the superior semicircular canal dehiscence of 37 cadaver temporal bones. Surg Radiol.

[6] Benamira LZ, Alzahrani M, Saliba I (2014). Superior canal dehiscence: can we predict the diagnosis?. Otol Neurotol.

[7] Janky KL, Nguyen KD, Welgampola M, Zuniga MG, Carey JP (2013). Air-conducted oVEMPs provide the best separation between intact and superior canal dehiscent labyrinths. Otol Neurotol.

[8] Chien WW, Carey JP, Minor LB (2011). Canal dehiscence. Curr Opin Neurol.

[9] Niesten MEF, McKenna MJ, Herrmann BS, Grolman W, Lee DJ (2013). Utility of cVEMPs in bilateral superior canal dehiscence syndrome. Laryngoscope.

[10] Zuniga MG, Janky KL, Nguyen KD, Welgampola MS, Carey JP (2013). Ocular versus cervical VEMPs in the diagnosis of superior semicircular canal dehiscence syndrome. Otol Neurotol.

[11] Kantner C, Gürkov R (2012). Characteristics and clinical applications of ocular vestibular evoked myogenic potentials. Hear Res.

[12] Agrawal SK, Parnes LS (2008). Transmastoid superior semicircular canal occlusion. Otol Neurotol.

[13] Zhao Zhao YC, Somers T, van Dinther J, Vanspauwen R, Husseman J, Briggs R (2012). Transmastoid repair of superior semicircular canal dehiscence. Skull Base.

[14] Beyea JA, Agrawal SK, Parnes LS (2012). Transmastoid semicircular canal occlusion: a safe and highly effective treatment for benign paroxysmal positional vertigo and superior canal dehiscence. Laryngoscope.

[15] Amoodi HA, Makki FM, McNeil M, Bance M (2011). Transmastoid resurfacing of superior semicircular canal dehiscence. Laryngoscope.

[16] de Bruijn AJ, Tange RA, Dreschler WA (2001). Efficacy of evaluation of audiometric results after stapes surgery in otosclerosis. II. A method for reporting results from individual cases. Otolaryngol Head Neck Surg.

[17] Mikulec AA, Poe DS, McKenna MJ (2005). Operative management of superior semicircular canal dehiscence. Laryngoscope.

[18] Ward BK, Agrawal Y, Nguyen E (2012). Hearing outcomes after surgical plugging of the superior semicircular canal by a middle cranial fossa approach. Otol Neurotol.

[19] Minor LB (2005). Clinical manifestations of superior semicircular canal dehiscence. Laryngoscope.

[20] Phillips DJ, Souter MA, Vitkovic J, Briggs RJ (2010). Diagnosis and outcomes of middle cranial fossa repair for patients with superior semicircular canal dehiscence syndrome. J Clin Neurosci.

[21] McCall AA, McKenna MJ, Merchant SN, Curtin HD, Lee DJ (2011). Superior canal dehiscence syndrome associated with the superior petrosal sinus in pediatric and adult patients. Otol Neurotol.

[22] Koo JW, Hong SK, Kim DK, Kim JS (2010). Superior semicircular canal dehiscence syndrome by the superior petrosal sinus. J Neurosurg.

[23] Carey JP, Minor LB, Nager GT (2000). Dehiscence or thinning of bone overlying the superior semicircular canal in a temporal bone survey. Arch Otolaryngol Head Neck Surg.

[24] Niesten ME, Hamberg LM, Silverman JB (2014). Superior canal dehiscence length and location influences clinical presentation and audiometric and cervical vestibular-evoked myogenic potential testing. Audiol Neurootol.

[25] Vlastarakos PV, Proikas K, Tavoulari E, Kikidis D, Maragoudakis P, Nikolopoulos TP (2009). Efficacy assessment and complications of surgical management for superior semicircular canal dehiscence: a meta-analysis of published interventional studies. Eur Arch Otorhinolaryngol.

[26] Hirvonen TP, Weg N, Zinreich SJ, Minor LB (2003). High-resolution CT findings suggest a developmental abnormality underlying superior canal dehiscence syndrome. Acta Otolaryngol.

[27] Aw ST, Aw GE, Todd MJ, Bradshaw AP, Halmagyi GM (2010). Three-dimensional vibration-induced vestibulo-ocular reflex identifies vertical semicircular canal dehiscence. JARO.

[28] Carey JP, Migliaccio AA, Minor LB (2007). Semicircular canal function before and after surgery for superior canal dehiscence. Otol Neurotol.

[29] Brantberg K, Bergenius J, Mendel L, Witt H, Tribukait A, Ygge J (2001). Symptoms, findings and treatment in patients with dehiscence of the superior semicircular canal. Acta Otolaryngol.

[30] Songer JE, Rosowski JJ (2010). A superior semicircular canal dehiscence-induced air-bone gap in chinchilla. Hear Res.

[31] Welgampola MS, Myrie OA, Minor LB, Carey JP (2008). Vestibular-evoked myogenic potential thresholds normalize on plugging superior canal dehiscence. Neurology.

[32] Yew A, Zarinkhou G, Spasic M, Trang A, Gopen Q, Yang I (2012). Characteristics and management of superior semicircular canal dehiscence. Skull Base.

[33] Janky KL, Zuniga MG, Carey JP, Schubert M (2012). Balance dysfunction and recovery after surgery for superior canal dehiscence syndrome. Arch Otolaryngol Head Neck Surg.

[34] Crane BT, Minor LB, Carey JP (2008). Superior canal dehiscence plugging reduces dizziness handicap. Laryngoscope.

[35] Bogle JM, Lundy LB, Zapala DA, Copenhaver A (2013). Dizziness handicap after cartilage cap occlusion for superior semicircular canal dehiscence. Otol Neurotol.

[36] Kim TH, Nam BH, Park CI (2002). Histologic changes of lateral semicircular canal after transection and occlusion with various materials in chinchillas. Korean J Otolaryngol.

[37] Darrouzet V, Duclos JY, Liguoro D, Truilhe Y, De Bonfils C, Bebear JP (2001). Management of facial paralysis resulting from temporal bone fractures: our experience in 115 cases. Otolaryngol Head Neck Surg.

[38] Crovetto M, Areitio E, Elexpuru J, Aguayo F (2008). Transmastoid approach for resurfacing of Superior Semicircular Canal dehiscence. Auris Nasus Larynx.

[39] Deschenes GR, Hsu DP, Megerian CA (2009). Outpatient repair of superior semicircular canal dehiscence via the transmastoid approach. Laryngoscope.

[40] Monsell E, Balkany T, Gates G, Goldenberg RA, Meyerhoff WL, House JW (1995). Committee on hearing and equilibrium guidelines for the evaluation of results of treatment of conductive hearing loss. Otolaryngol Head Neck Surg.

